# Association between immune cell attributes, serum metabolites, inflammatory protein factors, and colorectal cancer: A Mendelian randomization study

**DOI:** 10.1097/MD.0000000000040691

**Published:** 2024-11-29

**Authors:** Jingting Zhang, Hongyao Chen, Renyi Yang, Jincheng Tang, Xiaopeng Yu, Puhua Zeng, Wei Peng

**Affiliations:** a Hunan Provincial Hospital of Integrated Traditional Chinese and Western, Cancer Research Institute of Hunan Academy of Traditional Chinese Medicine, Hunan Academy of Chinese Medicine, Hunan, China; b School of Traditional Chinese Medicine, Hunan University of Chinese Medicine, Hunan, China.

**Keywords:** colorectal cancer, immune cell attributes, inflammatory protein factors, Mendelian randomization analysis, serum metabolites

## Abstract

Understanding the role of the tumor microenvironment in colorectal cancer (CRC) progression remains a challenge due to its complexity. Investigating the interplay between immune cell characteristics, serum metabolites, inflammatory protein factors, and CRC could unveil novel therapeutic avenues. We used 2-sample Mendelian randomization (MR) on Genome-Wide Association Studies (GWAS) data to explore causal links between 731 immune cell characteristics, 1400 serum metabolites, 91 inflammatory proteins, and CRC. Various MR methods, including inverse variance weighted (IVW) and MR-Egger, were applied to ensure robust analysis. Sensitivity analyses, such as the MR-Egger intercept test, Cochran’s *Q* test, and leave-one-out analysis, were performed to check for pleiotropy, heterogeneity, and influential outliers. Following rigorous genetic variation screening, we identified 43 immune cell characteristics associated with CRC. Notably, 7 immunophenotypes, including CD39^+^ CD4^+^ T cell Absolute Count, exhibited significant associations as protective factors. Additionally, 36 other immunophenotypes showed significant causal relationships with CRC. Among serum metabolites, 37 were correlated with CRC, with 1-arachidonoyl-gpc (20: 4n6) being the most closely linked as a risk factor. Similarly, 36 serum metabolites displayed significant causal relationships with CRC. Seven inflammatory protein factors exhibited causal relationships with CRC, with 4 posing as risk factors and 3 as protective factors. Our study scrutinized 731 immune cell characteristics, 1400 serum metabolites, and 91 inflammatory protein factors within the tumor microenvironment. We confirmed causal relationships between 43 immune cell characteristics, 37 serum metabolites, and 7 inflammatory protein factors with CRC. These findings offer novel insights into the potential etiology, prevention, and treatment strategies for CRC.

## 1. Introduction

The globally escalating incidence and mortality rate of cancer is cause for considerable concern. As delineated by the “Global Cancer Statistics 2020,”^[1]^ colorectal cancer (CRC) holds a prominent place with one of the highest incidence and mortality rates among cancers worldwide. Tumor microenvironment refers to the tissue environment around tumor cells, including tumor cells, microorganisms, extracellular matrix, blood vessels and immune cells. Tumor microenvironment plays an important role in the occurrence and development of tumors.^[2,3]^

Tumor cells employ a plethora of strategies to evade immune detection, thereby fostering an immunosuppressive environment. One critical interaction facilitating this evasion involves the programmed cell death 1 ligand 1 (PD-L1) and the programmed death receptor 1. This interaction, quintessential to the dialogue between tumor cells and immune cells, can drastically alter T cell activity. Specifically, the PD-L1/programmed death receptor 1 engagement leads to inhibitory outcomes, compromising T cell proliferation, survival, and cytokine production.^[4]^ Tumor cells elude immunological scrutiny and perpetuate their proliferation, thereby fostering the genesis and evolution of neoplasms. An abundance of biomarkers resides within serum metabolites, presenting potential targets for preliminary diagnosis or subsequent therapeutic strategies for cancer. For instance, a particular study identified the marked upregulation of methylmalonic acid within the serum of older individuals. Methylmalonic acid, a derivative of propionic acid metabolism, mediates neoplasm progression. Consequently, it’s postulated that metabolic alterations invoke a systemic environment fostering tumor progression.^[5]^ In recent times, an escalating number of studies have demonstrated that inflammatory protein constituents significantly contribute to the initiation and progression of neoplasms. It has been observed that inflammatory cells, accompanied by cytokines, potentially enhance tumor growth while facilitating the progression of immunosuppression. Furthermore, the proclivity and intensity of malignancies are intimately interconnected with inflammatory cytokines.^[6]^ Pertinent research underscores that tumor cells, under the inducement of hypoxia, are capable of generating inflammatory cytokines and chemokines. Such phenomena could potentially instigate direct advancement of the tumor progression.^[7]^

Nonetheless, the correlation between the tumor microenvironment and the development of colorectal cancer remains shrouded in numerous uncertainties. Although the relationship between the tumor microenvironment and CRC has been widely investigated, the limitations inherent in observational studies and animal experiments hinder the ability to establish causality. Mendelian randomization (MR) analysis, which uses genetic variants as instrumental variables, offers a way to overcome confounding and reverse causality, providing more robust evidence for causal inference. In recent years, MR analysis has been extensively applied to investigate the pathogenesis of various neoplastic diseases. Notably, MR studies have explored the causal relationships between gut microbiota and tumors, revealing that specific microbial species,^[8]^ such as Anaplasma krebsiensis, are associated with an increased risk of lung cancer. Furthermore, it was found that higher levels of cystine and propionylcarnitine are associated with a reduced risk of lung cancer.^[9]^ This study will focus on the immune cell profiles, serum metabolites, and inflammatory protein factors within the tumor microenvironment, investigating their causal relationship with CRC using MR analysis. While previous studies have established causal links between these factors and other diseases,^[10–12]^ there is currently no literature exploring their association with CRC. From the backdrop of these uncertainties, our study strategically selected 3 factors intricately associated with the tumor microenvironment: immune cell properties, serum metabolites, and inflammatory protein factors. An in-depth MR analysis was subsequently conducted to scrutinize any potential causal relationships between these 3 aspects and colorectal cancer. This exploration not only further elucidates the mechanistic processes involved in the inception and progression of colorectal cancer but also carves out a novel trajectory for its prevention and treatment.

## 2. Materials and methods

### 2.1. Research design

**Figure 1.**
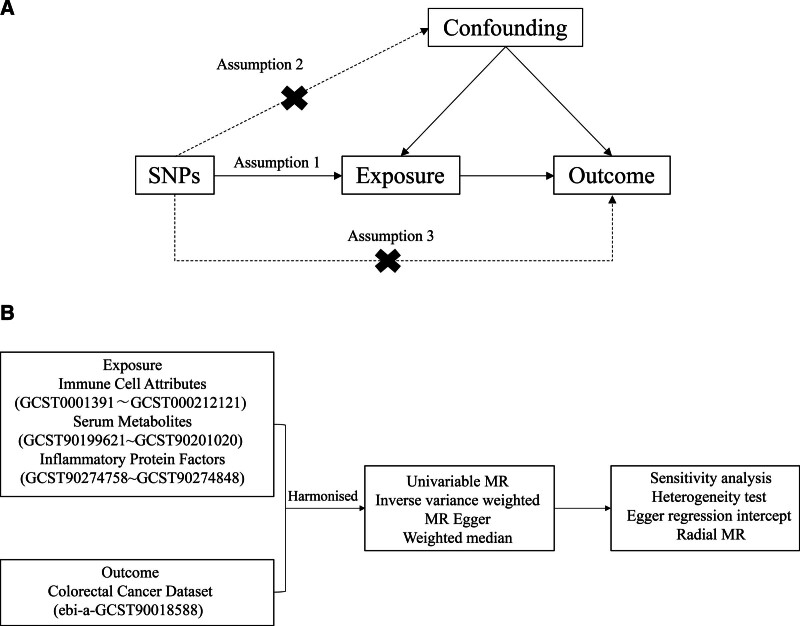
(A) Conceptual framework diagram for Mendelian randomization analysis. (B) Flow diagram of Mendelian randomization. MR = Mendelian randomization, SNP = single-nucleotide polymorphism.

We evaluated the causal relationship between 731 immune cell characteristics, 1400 serum metabolites, and 91 inflammatory protein factors and colorectal cancer based on a 2-sample MR analysis. MR analysis employs genetic variance as surrogates for risk determinants (Fig. 1). Consequently, efficacious instrumental variables (IVs) fulfilling 3 fundamental prerequisites are indispensably required for precise causal inference: The Relevance Postulate, implying that genetic variants have a direct association with the exposure variables; The Independence Axiom, meaning a state of independence exists between potential confounders related to genetic variation, exposure, and outcome; The Exclusivity Proposition, asserting that the impact of genetic variance on the outcome operates solely through the exposure.

### 2.2. Exposure factors

Single-nucleotide polymorphisms (SNPs) that demonstrated significant associations with immune cells, serum metabolites, and inflammatory protein factors were judiciously selected as instrumental variables. Summary statistics for Genome-Wide Association Studies (GWAS) concerning traits of immune cells are readily accessible within the public domain via the GWAS catalog.^[12,13]^ This expansive data set includes 731 immune-related tables (GCST0001391~GCST000212121). Specifically, it encapsulates data related to: median fluorescence intensity (MFI), relative cell counts (RC), absolute cell counts (AC), as well as morphological parameters (MP). The GWAS catalogue provides a public compilation of summary statistics for serum metabolites, encompassing a total of 1400 distinct types of serum metabolites (GCST90199621~GCST90201020).^[10]^ Stemming from the Canadian Longitudinal Study of Aging (CLSA), this study comprises genome-wide genotyping data of 8299 unrelated European subjects, along with analytic results of circulating plasma metabolites. In addition, the GWAS catalogue also offers publicly accessible summary statistics of inflammatory protein factors, with 91 distinct categories of these factors (GCST90274758~GCST90274848) mapped. The dataset was consolidated over 11 cohorts, encompassing a total of 14,824 participants. It incorporated the Olink Target Inflammation panel to measure the genome-wide gene data as well as plasma proteomics data pertaining to these individuals.^[11]^

In the bi-directional Mendelian randomization study of immune cells and serum metabolites, we employed significant genome-wide SNPs (*P* < 5 × 10^−8^)^[14]^ as inclusion criteria in an effort to discern more compelling causal associations. To broaden the investigation, thus capturing a more comprehensive picture of the relationships between cancer and immune cell populations, we adopted a secondary threshold for SNP identification. Specifically, SNPs falling at significance levels below 1 × 10^−6[15]^ were selected. This approach was intended to uncover a wider gamut of potential causal associations. To mitigate the bias caused by linkage disequilibrium (LD), we introduced 2 values: *r*² and kilobases (kb). The *r*² value indicates the degree of LD between 2 loci, ranging from 0 to 1. A higher *r*² suggests stronger LD between the loci, meaning that the genetic variations at these sites are more likely to be inherited together. The kb value represents the physical distance between 2 loci, measured in base pairs. In Mendelian Randomization analyses, researchers often set a high kb threshold, such as kb = 10,000, meaning that only when the physical distance between 2 loci exceeds 10,000 base pairs are they considered to have no significant LD, and can thus be treated as independent instrumental variables for analysis. Based on this and referencing previous studies,^[9]^ we set *r*² = 0.001 and kb = 10,000 as thresholds to eliminate linkage disequilibrium, selecting only the SNPs with the strongest effect on the outcome as instruments.

### 2.3. Outcome dataset

The colorectal cancer dataset was retrieved from the Open GWAS database of the Integrative Epidemiology Unit (IEU; https://gwas.mrcieu.ac.uk/), specifically, the ebi-a-GCST90018588 dataset. This comprehensive dataset comprises data from 167,691 samples, including 8305 cases from the tumor group and 159,386 from the normal control group. Furthermore, it encompasses in-depth information on 12,453,143 SNPs. We harnessed the identified SNPs of genome-wide significance (*P* < 5 × 10^−8^) as a criterial sieve for unearthing further potential causal associations. Concurrently, for mitigating the offset instigated by LD, we established a threshold of *r*^2^ = 0.001 and kb = 10000 to exclude LD. Precisely those SNPs imparting paramount effects on outcomes were cherry-picked as instruments. Acquisition of this preliminary study was executed with comprehended consent from all participants, and the encompassing data are ubiquitously accessible via the dedicated website.

### 2.4. MR analysis

This research employs the IVW approach,^[16]^ a method fundamentally grounded in meta-analysis principles, as its primary analytical tool to evaluate the peril interplay between exposure factors and ensuing outcomes. This method, aimed at deriving a comprehensive estimate of the exposure’s effect on results, is substantively transformed into a weighted regression analysis, focusing on the influence of instrumental factors on the outcomes of exposure effects. IVW can obviate confounding factors, negating the impact of horizontal pleiotropy, and thus produce undistorted estimations. To account for possible horizontal pleiotropy, we also employ 2 supplementary methodologies. However, it should be noted that these have comparatively diminished statistical power relative to the IVW approach: The weighted median (WM) method,^[17]^ and the MR-Egger method.^[18]^

Should the *P* value be <.05, statistically significant differences are inferred. An *F* value is designated as being >10 to circumvent the bias of weak instrumental variables, consequently implying the absence of such bias. The calculation formula for this is *F* = *R*^2^(N − 2)/(1 − *R*^2^), wherein *R*^2^ represents the variance proportion explained by the SNPs within the exposure database, and N indicates the sample size of the GWAS exposure. Colorectal cancer, defined binary, serves as the outcome, and is represented as both an odds ratio (OR) and a 95% confidence interval (CI). R4.2.3 and R studio software and R package ‘TwoSampleMR’ were used for the above analysis, and the test standard was α = 0.05.

### 2.5. Sensitivity analysis

As per Mendelian randomization, genetic tools can influence outcomes solely through individual exposure, acknowledging that genetic variations might display pleiotropy. In our principal analysis, we compute Wald ratio approximations for each genetic variant, amassing these calculations via the IVW technique. The IVW methodology integrated with multiplicative random effects furnishes a succinct estimate, while accommodating potential heterogeneity amid the Wald ratio appraisals of SNPs. However, if the SNPs utilized as instruments exhibit horizontal pleiotropic implications, which thereby influence results through pathways extrinsic to the exposure, the estimation might be compromised in accuracy. The Cochran’s *Q* test was utilized to evaluate potential heterogeneity. Statistically, a value of *P* > .05 indicated an absence of heterogeneity amongst SNPs, thereby justifying the use of a fixed-effect IVW model. Conversely, should *P* < .05, it would suggest the presence of heterogeneity amongst SNPs. In such a scenario, the implementation of a random-effects IVW model would be deemed appropriate.^[19]^ Utilizing the Leave-one-out analysis approach, we estimated the sensitivity of impact evaluation as individual SNPs were sequentially eliminated. This was performed to assess the influence of removing a single SNP observation on the ultimate outcome. The intercept generated through the application of MR Egger regression analysis served as an evaluative measure for potential pleiotropic effects. A resulting *P > *.05 signified an absence of such pleiotropy.

### 2.6. Statistical analysis

In the aforementioned analysis, we utilized the R software (version 4.2.0, available at http://www.r-project.org) in conjunction with the “TwoSampleMR” package (version 0.5.6).^[20]^

## 3. Results

### 3.1. The causal relationship between immunocyte characteristics and the colorectal cancer

In the MR analysis pertaining to immune cell characteristics and colorectal cancer, we observed that the count of SNPs integrated into CD25 on CD39^+^ activated CD4 regulatory T cells (ebi-a-GCST90001940), and CD25 on CD39^+^ CD4 regulatory T cells (ebi-a-GCST90001935), was <3. Although yielding a significant of IVW (*P* = .026, 0.032 < 0.05), their repercussions remained unevaluatable. Hence, this avenue was deemed unsuitable for further examination and was thus disregarded.

**Figure 2.**
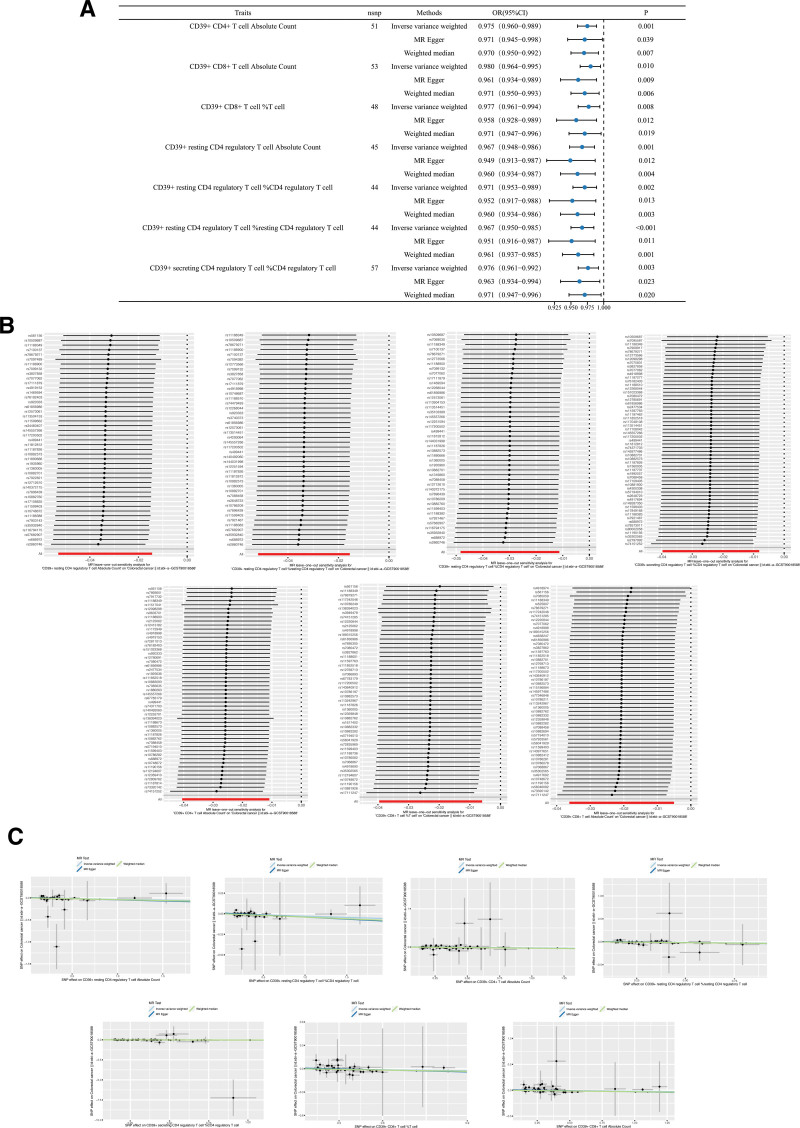
Mendelian randomization analysis of immune cell characteristics with CRC. (A) Forest plot of significance of MR analysis for 7 strong positive exposures. (B) Leave-one-out plot of sensitivity analysis for 7 strong positive exposures. (C) Funnel plot of different MR analyses for 7 strong positive exposures. CRC = colorectal cancer, MR = Mendelian randomization.

We subsequently ascertained that there are 7 exposure factors demonstrated a significant causal association with colorectal cancer, including CD39^+^ CD4^+^ T cell absolute count (ebi-a-GCST90001660; IVW: OR [95%CI] = 0.975 [0.960–0.989], *P* = .001; MR Egger: OR [95%CI] = 0.971 [0.945–0.998], *P* = .039; weighted median: OR [95%CI] = 0.970 [0.950–0.992], *P* = .007), CD39^+^ CD8^+^ T cell absolute count (ebi-a-GCST90001672; IVW: OR [95%CI] = 0.980 [0.964–0.995], *P* = .010; MR Egger: OR [95%CI] = 0.961 [0.934–0.989], *P* = .009; weighted median: OR [95%CI] = 0.971 [0.950–0.993], *P* = .006), CD39^+^ CD8^+^ T cell %T cell (ebi-a-GCST90001670; IVW: OR [95%CI] = 0.977 [0.961–0.994], *P* = .008; MR Egger: OR [95%CI] = 0.958 [0.928–0.989], *P* = .012; weighted median: OR [95%CI] = 0.971 [0.947–0.996], *P* = .019), et al. Moreover, these results demonstrate robustness. As such, the 7 aforementioned exposure factors serve as protective elements against colorectal cancer (Fig. 2).

**Figure 3.**
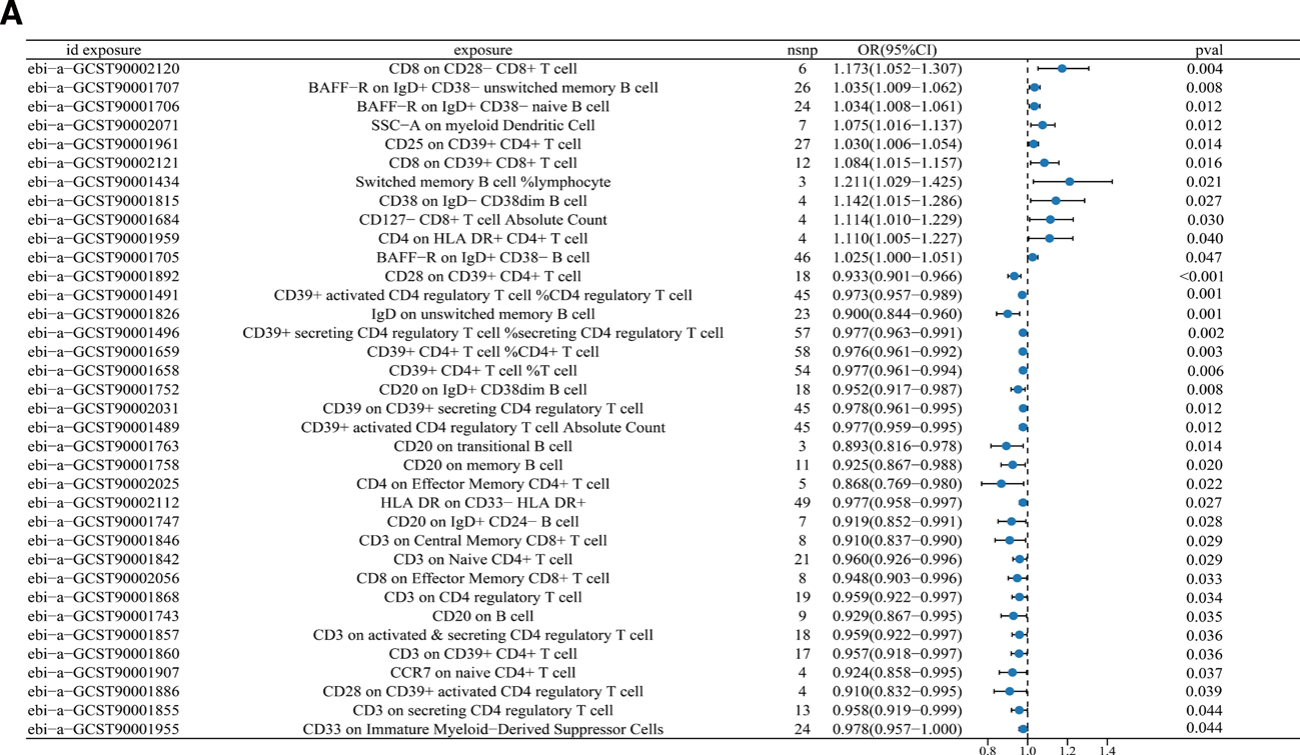
(A) Forest plot of other exposures of immune cell characteristics with colorectal under IVW. IVW = inverse variance weighted.

Utilizing the IVW approach as the primary analytical tool, we identified including CD8 on CD28^−^CD8^+^ T cell (ebi-a-GCST90002120; IVW: OR [95%CI] = 1.173 [1.0521.307], *P* = .004), BAFF-R on IgD^+^ CD38^−^ unswitched memory B cell (ebi-a-GCST90001707; IVW: OR [95%CI] = 1.035 [1.0091.061], *P* = .008). BAFF-R on IgD^+^ CD38^−^ naive B cell (ebi-aGCST90001706; IVW: OR [95%CI] = 1.034 [1.007–1.061], *P* = .012), etc, 11 immune cell attributes correlated with colorectal cancer (Fig. 3). These attributes function as potential risk factors for the aforementioned disease. Moreover, our investigation revealed an association between colorectal cancer and 25 immune cell attributes (Fig. 3). Intriguingly, these signatures function as protective factors in the context of colorectal cancer, including CD28 on CD39^+^ CD4^+^ T cell (ebi-a-GCST90001892; IVW: OR [95%CI] = 0.933 [0.901–0.966], *P* < .001), CD39^+^ activated CD4 regulatory T cell %CD4 regulatory T cell (ebi-a-GCST90001491; IVW: OR [95%CI] = 0.973 [0.957–0.989], *P* = .001), IgD on unswitched memory B cell (ebi-a-GCST90001826; IVW: OR [95%CI] = 0.900 [0.844–0.960], *P* = .001), et al. The robustness of the above exposure factors is second.

A Cochran *Q* test was employed to examine the potential heterogeneity across these exposure factors, revealing no considerable heterogeneity (*Q_pval* > 0.05) (Table 1). Consequently, a fixed effect IVW model was adopted. Furthermore, an intercept test under the MR-Egger regression framework was utilized to assess potential pleiotropy among the aforementioned exposure factors, indicating no suggestive evidence of pleiotropic effects (*P* > .05; Table 1).

**Table 1:** Heterogeneity tests and directional horizontal pleiotropy of MR results between immune cell characteristics with CRC.

id.exposure	Exposure	Heterogeneity tests		Directional horizontal pleiotropy
		MR Egger	Inverse variance weighted	egger_intercept
		Q_pval	Q_pval	pval
ebi-a-GCST90001705	BAFF-R on IgD^+^ CD38^−^ B cell	0.159	0.184	0.839
ebi-a-GCST90001706	BAFF-R on IgD^+^ CD38^−^ naive B cell	0.165	0.197	0.700
ebi-a-GCST90001707	BAFF-R on IgD^+^ CD38^−^ unswitched memory B cell	0.190	0.208	0.490
ebi-a-GCST90001907	CCR7 on naive CD4^+^ T cell	0.635	0.721	0.581
ebi-a-GCST90001857	CD3 on activated and secreting CD4 regulatory T cell	0.698	0.702	0.367
ebi-a-GCST90001868	CD3 on CD4 regulatory T cell	0.714	0.727	0.413
ebi-a-GCST90001860	CD3 on CD39^+^ CD4^+^ T cell	0.785	0.676	0.140
ebi-a-GCST90001846	CD3 on central memory CD8^+^ T cell	0.469	0.167	0.071
ebi-a-GCST90001842	CD3 on Naive CD4^+^ T cell	0.173	0.207	0.685
ebi-a-GCST90001855	CD3 on secreting CD4 regulatory T cell	0.499	0.502	0.348
ebi-a-GCST90002025	CD4 on Effector Memory CD4^+^ T cell	0.199	0.322	0.893
ebi-a-GCST90001959	CD4 on HLA DR^+^ CD4^+^ T cell	0.806	0.894	0.712
ebi-a-GCST90002120	CD8 on CD28^−^ CD8^+^ T cell	0.934	0.959	0.671
ebi-a^-^GCST90002121	CD8 on CD39^+^ CD8^+^ T cell	0.538	0.531	0.328
ebi-a-GCST90002056	CD8 on effector memory CD8^+^ T cell	0.246	0.342	0.997
ebi-a-GCST90001743	CD20 on B cell	0.773	0.848	0.854
ebi-a-GCST90001747	CD20 on IgD^+^ CD24^−^ B cell	0.466	0.455	0.338
ebi-a-GCST90001752	CD20 on IgD^+^ CD38dim B cell	0.568	0.533	0.246
ebi-a-GCST90001758	CD20 on memory B cell	0.456	0.523	0.601
ebi-a-GCST90001763	CD20 on transitional B cell	0.677	0.868	0.797
ebi-a-GCST90001961	CD25 on CD39^+^ CD4^+^ T cell	0.314	0.351	0.617
ebi-a-GCST90001886	CD28 on CD39^+^ activated CD4 regulatory T cell	0.578	0.747	0.752
ebi-a-GCST90001892	CD28 on CD39^+^ CD4^+^ T cell	0.929	0.948	0.735
ebi-a-GCST90001955	CD33 on immature myeloid-derived suppressor cells	0.899	0.742	0.052
ebi-a-GCST90001815	CD38 on IgD^−^ CD38dim B cell	0.998	0.778	0.405
ebi-a-GCST90001489	CD39^+^ activated CD4 regulatory T cell absolute count	0.852	0.876	0.981
ebi-a-GCST90001491	CD39^+^ activated CD4 regulatory T cell %CD4 regulatory T cell	0.938	0.950	0.838
ebi-a-GCST90001660	CD39^+^ CD4^+^ T cell absolute count	0.507	0.544	0.746
ebi-a-GCST90001659	CD39^+^ CD4^+^ T cell %CD4^+^ T cell	0.894	0.911	0.928
ebi-a-GCST90001658	CD39^+^ CD4^+^ T cell %T cell	0.802	0.826	0.760
ebi-a-GCST90001672	CD39^+^ CD8^+^ T cell absolute count	0.627	0.569	0.124
ebi-a-GCST90001670	CD39^+^ CD8^+^ T cell %T cell	0.869	0.837	0.160
ebi-a-GCST90002031	CD39 on CD39^+^ secreting CD4 regulatory T cell	0.901	0.919	0.910
ebi-a-GCST90001483	CD39^+^ resting CD4 regulatory T cell Absolute Count	0.794	0.786	0.295
ebi-a-GCST90001485	CD39^+^ resting CD4 regulatory T cell %CD4 regulatory T cell	0.989	0.986	0.243
ebi-a-GCST90001484	CD39^+^ resting CD4 regulatory T cell %resting CD4 regulatory T cell	0.968	0.965	0.312
ebi-a-GCST90001497	CD39^+^ secreting CD4 regulatory T cell %CD4 regulatory T cell	0.721	0.720	0.335
ebi-a-GCST90001496	CD39^+^ secreting CD4 regulatory T cell %secreting CD4 regulatory T cell	0.933	0.944	0.746
ebi-a-GCST90001684	CD127^−^ CD8^+^ T cell absolute count	0.726	0.647	0.420
ebi-a-GCST90002112	HLA DR on CD33^−^ HLA DR^+^	0.015	0.007	0.079
ebi-a-GCST90001826	IgD on unswitched memory B cell	0.290	0.284	0.317
ebi-a-GCST90002071	SSC-A on myeloid dendritic cell	0.872	0.935	0.982
ebi-a-GCST90001434	Switched memory B cell %lymphocyte	0.165	0.247	0.623

CRC = colorectal cancer, MR = Mendelian randomization.

### 3.2. The causal relationship between serum metabolites and the colorectal cancer

In our pursuit to elucidate the causative impact of serum metabolites on colorectal cancer, a 2-sample Mendelian randomization analysis was leveraged. Our findings reveal that Arginine levels (GCST90200372), Arginine to phosphate ratio (GCST90200854) and X-11315 levels (GCST90200458) were each encapsulated in fewer than 3 SNPs given the predetermined conditions. Despite the statistical validity of the IVW (*P* < .05), its efficacy remains undetermined. Consequently, it was deemed necessary to discount these results.

**Figure 4.**
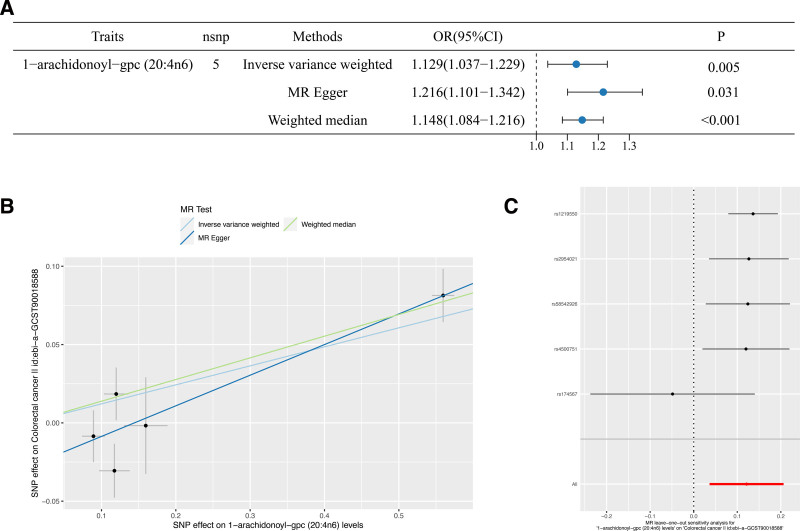
Mendelian randomization analysis of serum metabolites with CRC. (A) forest plot of significance of MR analysis for GCST9019978. (B) leave-one-out plot of sensitivity analysis for GCST90199788. (C) Funnel plot of different MR analyses for GCST90199788. CRC = colorectal cancer, MR = Mendelian randomization.

We found 1-arachidonoyl-gpc (20:4n6; GCST90199788; IVW: OR [95%CI] = 1.129 [1.037–1.229], *P* = .005; MR Egger: OR [95%CI] = 1.216 [1.101–1.342], *P* = .031; weighted median: OR [95%CI] = 1.148 [1.084–1.216], *P* < .001), which has a compelling evidence underpining the strong causal relationship with colorectal cancer, and it is a risk factor for colorectal cancer (Fig. 4).

**Figure 5.**
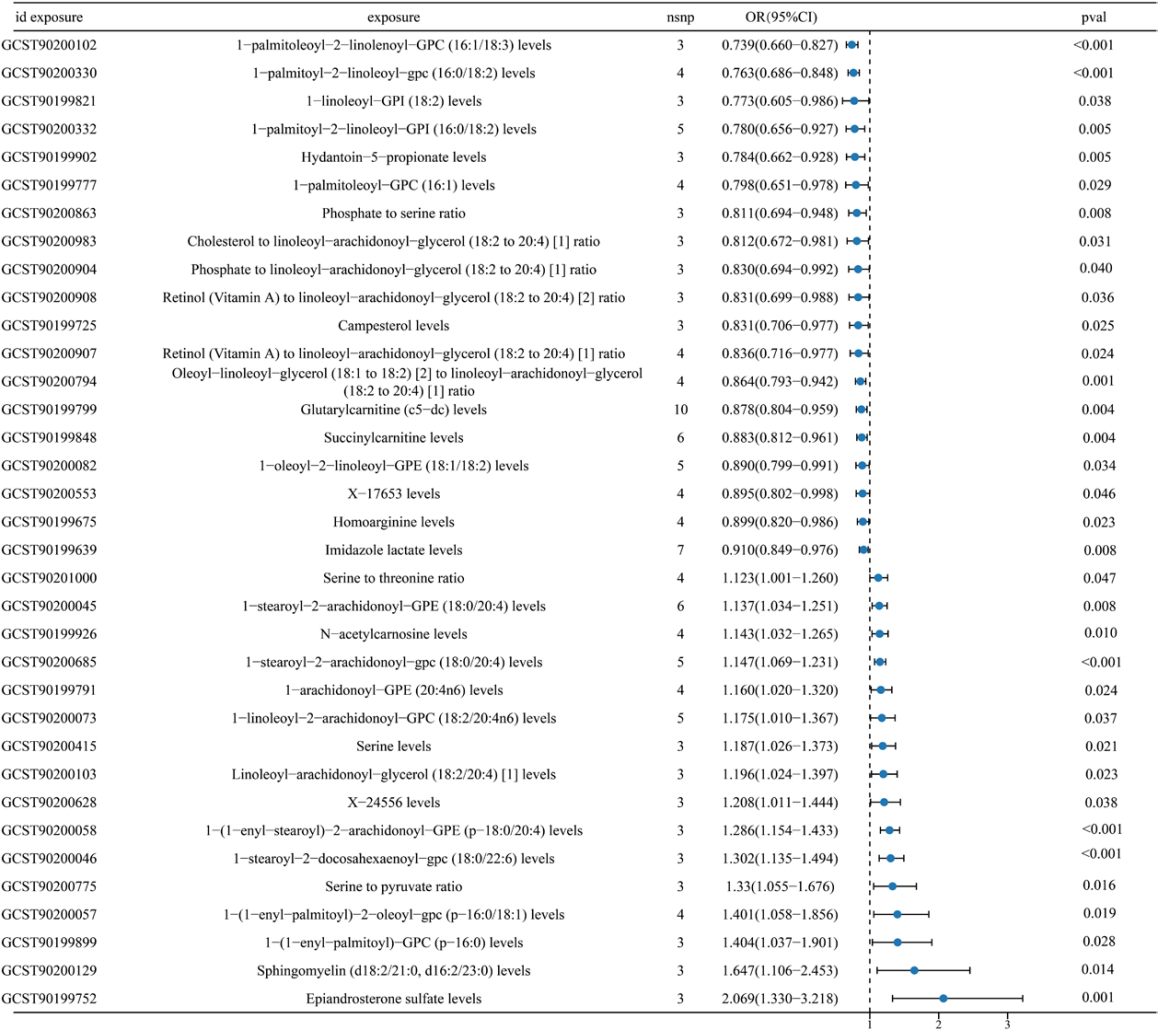
Forest plot of other exposures of serum metabolites with colorectal under IVW. IVW = inverse variance weighted.

When the IVW method was used as the primary method of analysis, we found that the 16 results including Epiandrosterone sulfate levels (GCST90199752; IVW: OR [95%CI] = 2.069 [1.330–3.218], *P* = .001), Sphingomyelin (d18:2/21:0, d16:2/23:0) levels (GCST90200129; IVW: OR [95%CI] = 1.647 [1.106–2.453], *P* = .014), 1-(1-enyl-palmitoyl)-GPC (p-16:0) levels (GCST90199899; IVW: OR [95%CI] = 1.404 [1.037–1.901], *P* = .028), etc, have been associated with colorectal cancer as a risk factor for the development of colorectal cancer. In addition, including 1-palmitoleoyl-2-linolenoyl-GPC (16:1/18:3) levels (GCST90200102; IVW: OR [95%CI] = 0.739 [0.660–0.827], *P* < .001), 1-palmitoyl-2-linoleoyl-gpc (16:0/18:2) levels (GCST90200330; IVW: OR [95%CI] = 0.763 [0.686–0.848], *P* < .001), 1-linoleoyl-GPI (18:2) levels (GCST90199821; IVW: OR [95%CI] = 0.773 [0.605–0.986], *P* = .038), etc, 19 serum metabolites were associated with colorectal cancer, and were protective factors against colorectal cancer, with the above exposures having a lower robustness (Fig. 5).

Furthermore, we employed the Cochran *Q* test to evaluate the potential heterogeneity of the aforementioned exposure factors. We observed 13 exposure factors that exhibited significant heterogeneity (*Q_pval* < 0.05), including 1-arachidonoyl-GPE (20:4n6) levels (GCST90199791), 1-stearoyl-2-arachidonoyl-GPE (18:0/20:4) levels (GCST90200045), 1-linoleoyl-2-arachidonoyl-GPC (18:2/20:4n6) levels (GCST90200073), et al. As a consequence, we resorted to the random effect inverse variance weighted (IVW) model for further analysis. There was no heterogeneity in the 22 exposure factors (*Q_pval* > 0.05), including 1-arachidonoyl-gpc (20:4n6) levels (GCST90199788), 1-palmitoyl-2-linoleoyl-GPI (16:0/18:2) levels (GCST90200332), 1-stearoyl-2-arachidonoyl-gpc (18:0/20:4) levels (GCST90200685), et al, so the fixed effect IVW model was selected. The potential pleiotropy of the above exposures was assessed by the intercept of the MR-Egger regression test, and there was no pleiotropy of any of the above exposures (i.e., *P* > .05; Table 2).

**Table 2:** Heterogeneity tests and directional horizontal pleiotropy of MR results between serum metabolites with CRC.

id.exposure	Exposure	Heterogeneity tests		Directional horizontal pleiotropy
		MR Egger	Inverse variance weighted	egger_intercept
		Q_pval	Q_pval	pval
GCST90199791	1-arachidonoyl-GPE (20:4n6) levels	0.004	0.011	0.906
GCST90200045	1-stearoyl-2-arachidonoyl-GPE (18:0/20:4) levels	0.006	0.012	0.98
GCST90200073	1-linoleoyl-2-arachidonoyl-GPC (18:2/20:4n6) levels	0.181	0.021	0.136
GCST90199821	1-linoleoyl-GPI (18:2) levels	0.721	0.025	0.226
GCST90200904	Phosphate to linoleoyl-arachidonoyl-glycerol (18:2 to 20:4) [1] ratio	0.988	0.029	0.228
GCST90199899	1-(1-enyl-palmitoyl)-GPC (p-16:0) levels	0.061	0.032	0.507
GCST90200082	1-oleoyl-2-linoleoyl-GPE (18:1/18:2) levels	0.043	0.033	0.422
GCST90200908	Retinol (vitamin A) to linoleoyl-arachidonoyl-glycerol (18:2 to 20:4) [2] ratio	0.869	0.033	0.233
GCST90200983	Cholesterol to linoleoyl-arachidonoyl-glycerol (18:2 to 20:4) [1] ratio	0.911	0.037	0.237
GCST90200907	Retinol (vitamin A) to linoleoyl-arachidonoyl-glycerol (18:2 to 20:4) [1] ratio	0.102	0.038	0.325
GCST90200057	1-(1-enyl-palmitoyl)-2-oleoyl-gpc (p-16:0/18:1) levels	0.209	0.041	0.211
GCST90200103	Linoleoyl-arachidonoyl-glycerol (18:2/20:4) [1] levels	0.712	0.045	0.245
GCST90200129	Sphingomyelin (d18:2/21:0, d16:2/23:0) levels	0.101	0.045	0.458
GCST90199788	1-arachidonoyl-gpc (20:4n6) levels	0.252	0.055	0.146
GCST90200332	1-palmitoyl-2-linoleoyl-GPI (16:0/18:2) levels	0.049	0.095	0.885
GCST90200685	1-stearoyl-2-arachidonoyl-gpc (18:0/20:4) levels	0.111	0.118	0.473
GCST90200794	Oleoyl-linoleoyl-glycerol (18:1 to 18:2) [2] to linoleoyl-arachidonoyl-glycerol (18:2 to 20:4) [1] ratio	0.278	0.151	0.281
GCST90199752	Epiandrosterone sulfate levels	0.801	0.275	0.358
GCST90200046	1-stearoyl-2-docosahexaenoyl-gpc (18:0/22:6) levels	0.474	0.276	0.387
GCST90201000	Serine to threonine ratio	0.264	0.322	0.513
GCST90200058	1-(1-enyl-stearoyl)-2-arachidonoyl-GPE (p-18:0/20:4) levels	0.323	0.41	0.534
GCST90199848	Succinylcarnitine levels	0.427	0.571	0.957
GCST90199902	Hydantoin-5-propionate levels	0.548	0.664	0.622
GCST90199639	Imidazole lactate levels	0.58	0.694	0.783
GCST90199799	Glutarylcarnitine (c5-dc) levels	0.654	0.729	0.693
GCST90199725	Campesterol levels	0.758	0.762	0.625
GCST90199926	N-acetylcarnosine levels	0.708	0.78	0.592
GCST90200628	X-24556 levels	0.586	0.781	0.734
GCST90199777	1-palmitoleoyl-GPC (16:1) levels	0.688	0.785	0.629
GCST90200330	1-palmitoyl-2-linoleoyl-gpc (16:0/18:2) levels	0.728	0.799	0.601
GCST90199675	Homoarginine levels	0.749	0.861	0.716
GCST90200553	X-17653 levels	0.881	0.863	0.556
GCST90200775	Serine to pyruvate ratio	0.843	0.981	0.99
GCST90200863	Phosphate to serine ratio	0.982	0.985	0.891
GCST90200102	1-palmitoleoyl-2-linolenoyl-GPC (16:1/18:3) levels	0.911	0.991	0.956
GCST90200415	Serine levels	0.91	0.992	0.964

CRC = colorectal cancer, MR = Mendelian randomization.

### 3.3. The causal relationship between inflammatory protein factors and the colorectal cancer

In order to investigate the causal effect of inflammatory protein factors on colorectal cancer, we adopted the MR analysis with 2 samples, with the IVW method as the primary analytical approach.

**Figure 6.**
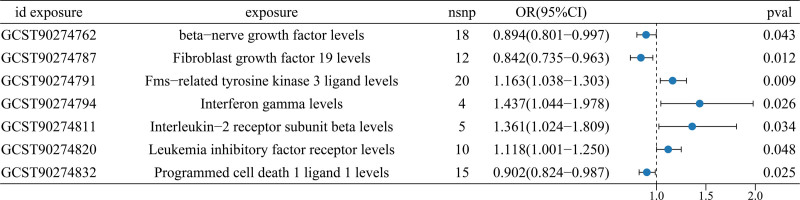
Forest plot of mendelian randomization analysis of inflammatory protein factors with CRC under IVW. CRC = colorectal cancer, IVW = inverse variance weighted.

**Figure 7.**
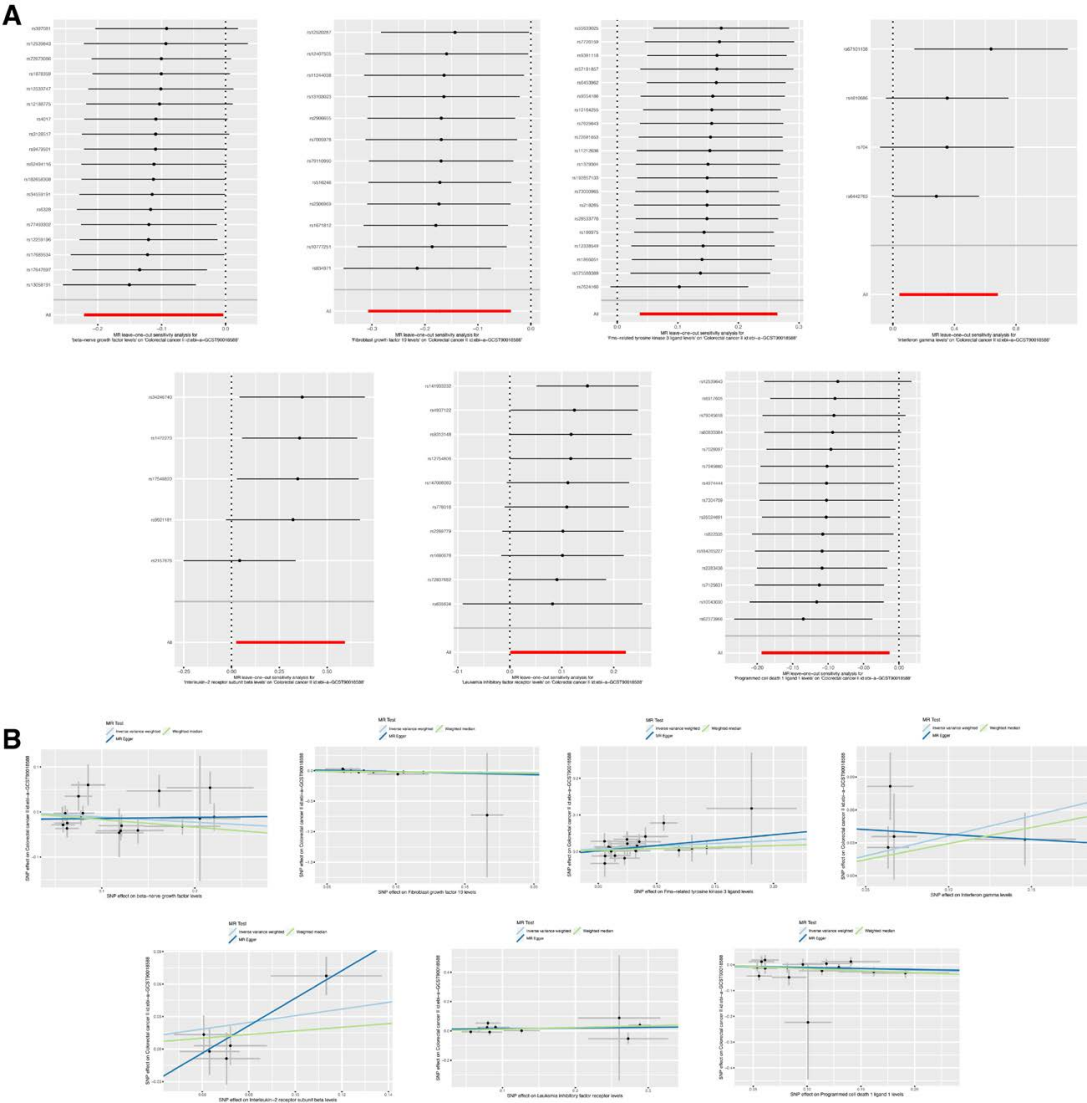
(A) Leave-one-out plot of sensitivity analysis for inflammatory protein factors. (B) Funnel plot of different MR analyses for inflammatory protein factors. MR = Mendelian randomization.

In our analysis examining the relationship between inflammatory protein factors and colorectal cancer, we identified 4 specific inflammatory protein factors, incorporating Interferon gamma levels (GCST90274794; IVW: OR [95%CI] = 1.437 [1.044–1.978], *P* = .026), interleukin-2 receptor subunit beta levels (GCST90274811; IVW: OR [95%CI] = 1.361 [1.024–1.809], *P* = .034), Fms-related tyrosine kinase 3 ligand levels (GCST90274791; IVW: OR [95%CI] = 1.163 [1.038–1.303], *P* = .009), Leukemia inhibitory factor receptor levels (GCST90274820; IVW: OR [95%CI] = 1.118 [1.001–1.250], *P* = .048),which exhibited a significant association with colorectal cancer, conferring it as perilous factors for this malignancy. Furthermore, programmed cell death 1 ligand 1 levels (GCST90274832; IVW: OR [95%CI] = 0.902 [0.824–0.987], *P* = .025) demonstrated an association with colorectal cancer. Similarly, measurements of betanerve growth factor levels (GCST90274762; IVW: OR [95%CI] = 0.894 [0.801–0.997], *P* = .043), as well as Fibroblast growth factor 19 levels (GCST90274787; IVW: OR [95%CI] = 0.842 [0.7350.963], *P* = .012), indicated their linkage to colorectal cancer. These 3 inflammatory protein factors are purported to act as protective agents against colorectal cancer (Figs. 6 and 7). These factors were the next most robust, with *P* < .05 when assessed by the IVW method only.

The Cochran *Q* test was implemented to evaluate the potential heterogeneity of the aforementioned exposure factors. No such heterogeneity was identified (*Q_pval* > 0.05; Table 3), thereby necessitating the selection of the fixed-effects IVW model. Furthermore, the intercept of the MR-Egger regression test was utilized to scrutinize potential multiplicity within the exposure factors. No multiplicity was discovered (*P* > .05; Table 3).

**Table 3:** Heterogeneity tests and directional horizontal pleiotropy of MR results between inflammatory protein factors with CRC.

id.exposure	Exposure	Heterogeneity tests		Directional horizontal pleiotropy
		MR Egger	Inverse variance weighted	egger_intercept
		Q_pval	Q_pval	pval
GCST90274762	Beta-nerve growth factor levels	0.280	0.244	0.230
GCST90274787	Fibroblast growth factor 19 levels	0.746	0.617	0.163
GCST90274791	Fms-related tyrosine kinase 3 ligand levels	0.337	0.364	0.476
GCST90274794	Interferon gamma levels	0.302	0.224	0.327
GCST90274811	Interleukin-2 receptor subunit beta levels	0.639	0.123	0.100
GCST90274820	Leukemia inhibitory factor receptor levels	0.151	0.166	0.451
GCST90274832	Programmed cell death 1 ligand 1 levels	0.384	0.454	0.779

CRC = colorectal cancer, MR = Mendelian randomization.

## 4. Discussion

From our results, a salient pattern emerged, revealing that 43 immunocyte attributes, 36 serum metabolites, and 7 inflammatory protein elements have significant causal associations with colorectal cancer.

The tumor microenvironment constitutes a complex ecosystem encompassing diverse cellular constituents, including but not limited to various immune cell types, tumor-related fibroblasts, and endothelial cells. This microenvironment is further enriched by an array of metabolites namely cytokines, chemokines, growth factors, and antibodies. These components may vary by tissue type and co-evolve with tumor progression. The immune system, emerging as a salient subject of extensive research within the realm of oncology, assumes a pivotal function within the tumor microenvironment. Extant investigations demonstrate that specific subgroups of tumor-associated myeloid cells within the tumor microenvironment uphold immunosuppressive properties. These cellular populations could potentially catalyze tumor progression by inhibiting immune cells, orchestrating stromal restructuring, and enhancing angiogenesis.^[21]^ Immune cells are both anti-tumor and pro-tumor immune. Investigations have revealed that human eosinophils are capable of inducing apoptosis and eradicating tumor cells present in the Colo - 205 colorectal cancer cell line. It has been proposed that granzyme A, a constituent of the eosinophilic protein pool, serves as a cytotoxic mediator, deploying its tumor-killing activity specifically against Colo - 205.^[22]^ In colorectal carcinoma observed in murine models, basophils have been demonstrated to play a dual role. They harbor the capability to instigate the infiltration of CD8^+^ T cells, thereby contributing to anti-tumor immunity.^[23]^ Conversely, they have also been observed to mitigate the influx of CD8^+^ T cells, subsequently promoting pro-tumor immunity.^[24]^ Cellular immune signatures could potentially modulate inflammatory responses, thereby impacting the progression of oncogenesis. Some tumor cells can secrete inhibitory mediators of T cells and natural killer cells, including interleukin 10, reactive oxygen species, transforming growth factor beta, and express immune checkpoint molecules such as PD-L1.^[25,26]^ And produce inflammatory mediators interleukin 1beta, interleukin 6 and other inflammatory factors to enhance inflammation.^[27]^ Employing SNPs, our findings divulged a potent association between 7 phenotypes of T cells and colorectal cancer, with each of them serving as protective factor, corroborated by an integration of multiple 2-sample MR. Concurrently, through the application of the IVW as the primary algorithm, and evaluating the heterogeneity and pleiotropy, we discerned an association between 36 immunophenotypes and colorectal cancer. Among these, 25 were identified as protective factors while 11 stood as risk factors.

One of the 10 major characteristics of tumor cells includes tumor-induced inflammation.^[28]^ Inflammation contributes to the acquisition of various hallmark functions by supplying bioactive molecules to the tumor microenvironment. An array of biochemical compounds discharging from the inflammatory cells, for example, reactive oxygen species, can precipitate the conversion of proximate cancerous cells into a highly malignant neoplastic state. We employing IVW as the primary algorithm and also performed a comprehensive evaluation of the heterogeneity and multifarious impact effect, which conclude that 4 inflammatory protein factors (i.e., fibroblast growth factor 19 levels, beta-nerve growth factor levels, programmed cell death 1 ligand 1 levels, Leukemia inhibitory factor receptor levels) are risk factors for colorectal cancer and 3 inflammatory protein factors (i.e., Fmsrelated tyrosine kinase 3 ligand levels, interleukin-2 receptor subunit beta levels, interferon gamma levels) are protective factors for colorectal cancer. A profusion of inflammatory cytokines permeates the tumor microenvironment, capable of not merely amplifying inflammatory responses, but also fostering tumor cell proliferation and metastasis. For example, IL-1α can determine the immunogenicity of chemically induced tumors and promote tumor-specific immune responses^[29]^; IL-12 can activate cytotoxic T cells, stimulate T cells and natural killer cells to produce IFN-γ, IL-2, TNF-α and other cytokines, promote the maturation and activation of DC and macrophages, and can feedback up-regulate the production of IL-12^[30]^; IL-17 can promote tumor cells to secrete pro-invasive factors to promote tumor invasion and metastasis^[31,32]^; IL-27 can induce CD8 ^+^ T cells to produce IFN-γ through STAT1-T-bet,^[33]^ thereby exerting anti-tumor activity. Fms-related tyrosine kinase 3 ligand may assist DCs in tumor antigen presentation and anti-tumor immune response. Low levels of Fms-related tyrosine kinase 3 ligand are associated with poor prognosis of tumors.^[34]^ A recent study has pointed out that Fibroblast growth factor 19 (FGR19) can induce angiogenesis, thereby promoting tumor progression.^[35]^

In the study of the causal relationship between serum metabolites and colorectal cancer, we integrated multiple 2-sample MR methods and found that 1-arachidonoyl-gpc (20: 4n6) was closely related to the occurrence of colorectal cancer and was a risk factor. 1-arachidonoyl-GPC (20:4n6), a product containing the FADS enzyme and omega-6 PUFA, was shown in the study by Mika et al to be preferentially incorporated and predominantly metabolized in colorectal cancer cells. These cells contain higher levels of both n-3 and n-6 PUFAs compared to normal intestinal mucosa. A potential explanation for this is that PUFAs are crucial for the formation of cell membrane phospholipids, a process essential for the rapid proliferation of cancer cells^[36]^; Similarly, Zhang et al^[37]^ reported elevated levels of n-6 PUFAs in the phospholipids of CRC tissues compared to the adjacent normal tissues. Arachidonic acid is an unsaturated fatty acid that acts as a phospholipid-bound structural lipid. Recently, through the data of human and mice, it has been proved from multiple dimensions that high content of AA will eventually promote tumor growth.^[38]^ These previous results are similar to those of the current study. When the IVW method was used as the main analysis method, we found that another 16 serum metabolites (e.g., serine levels) were risk factors for colorectal cancer, and 19 serum metabolites (e.g., homoarginine levels) were protective factors for colorectal cancer. Many tumors have metabolic alterations, including glucose metabolism, amino acid metabolism, nucleotide metabolism, fatty acid metabolism and lipid metabolism. Tryptophan is one of the essential amino acids for the human body. Studies have shown that tryptophan metabolites serotonin (5-HT) and 3-hydroxyanthranilic acid (3-HA) significantly inhibit ferroptosis of tumor cells, thereby inhibiting tumor progression.^[39]^ Serine is an important 1-carbon unit raw material, which can be obtained from the external environment through transporters or synthesized through the serine synthesis pathway as needed. Phosphoglycerate dehydrogenase (PHGDH) is an important rate-limiting enzyme in the endogenous serine synthesis pathway, which is highly expressed in various types of tumors (such as breast cancer, colorectal cancer, bladder cancer, etc). Serine is an important 1-carbon unit raw material, which can be obtained from the external environment through transporters or synthesized through the serine synthesis pathway as needed. Phosphoglycerate dehydrogenase (PHGDH) is an important rate-limiting enzyme in the endogenous serine synthesis pathway. It is highly expressed in various types of tumors (such as breast cancer, colorectal cancer, bladder cancer, etc), regulates serine metabolism and promotes tumor progression.^[40]^ Furthermore, arginine, as a precursor of polyamine metabolism, plays an important role in cell growth. Researchers have found that arginine content is increased in mouse models and human liver cancer cells, and arginine interacts with RNA-binding protein 39 (RBM39) to control the expression of metabolic genes. RBM39-mediated upregulation of aspartic acid synthesis leads to increased arginine uptake, forming a positive feedback loop to maintain high levels of arginine and tumor metabolism.^[41]^

In this study, we identified 43 immune cell characteristics, 37 serum metabolites, and 7 inflammatory protein factors that are causally associated with colorectal cancer (CRC) through Mendelian randomization analysis of 3 key indicators in the tumor microenvironment. These findings highlight the potential of immune cell profiles, serum metabolites, and inflammatory protein factors as biomarkers for early CRC diagnosis. For instance, our study demonstrated that higher absolute counts of CD39^+^ T cells are associated with a reduced risk of CRC, suggesting that the CD39^+^ T cell count may serve as a novel biomarker for early detection. Monitoring CD39^+^ T cell levels could improve early diagnosis rates and, in turn, patient prognosis. Moreover, the association of various immune cell profiles and serum metabolites with CRC risk indicates their potential utility in risk stratification, enabling more personalized prevention strategies and treatment plans. Additionally, we found that interferon-gamma and interleukin-2 receptor subunit β were linked to a reduced risk of CRC, suggesting that therapeutic agents targeting these molecules could be promising options in the treatment of colorectal cancer.

In the present research, we performed a 2-sample MR analysis, based on previously published results from a substantial GWAS cohort, which comes with several notable benefits. To begin with, due to the random assembly of alleles into gametes during meiosis, potential confounding elements cannot distort the causative link between genotype and disease within MR analysis. This major confounding issue flags other traditional observational studies. Furthermore, MR analysis benefits from being more accessible to the public compared to prospective cohort studies or randomized controlled trials, thereby reducing not only time, but also research cost. Lastly, our large sample size leads to high statistical efficiency. However, the present study is not without its bounds. Firstly, despite conducting numerous sensitivity analyses, we weren’t able to completely evaluate horizontal multiplicity. Given the differences among the included GWAS cohorts, such as variations in age, gender, and geographic location, the observed heterogeneity may reflect the influence of these factors on our results. This highlights the need for future studies to account for such confounding factors and employ more rigorous methods to control for them. Additionally, since our study primarily involved East Asian and European populations, the generalizability of our findings to other ethnic groups may be limited. Future research should include a more diverse range of populations to validate these results. Moreover, to incorporate more inflammatory factors in the MR analysis, we applied less stringent thresholds, which allowed for a more comprehensive assessment of the strong association between inflammatory factor profiles and cancer. However, this approach may have increased the likelihood of false positives. Thus, our findings should be interpreted with caution. Future research should utilize more advanced techniques to assess and control for multicollinearity to enhance the robustness of the results.

## 5. Conclusion

Our findings indicate robust associations between diverse immune cells, serum metabolites, inflammatory protein factors, and colorectal cancer. These findings warrant further investigation to validate these associations and explore the underlying mechanisms. Future research could focus on experimental studies to investigate the impact of modulating immune cell populations, serum metabolites, and inflammatory protein factors on colorectal cancer development and progression. Additionally, clinical studies are needed to assess the potential of these factors as prognostic or therapeutic targets for colorectal cancer.

## Author contributions

**Conceptualization:** Jingting Zhang.

**Data curation:** Jingting Zhang.

**Funding acquisition:** Puhua Zeng, Wei Peng.

**Investigation:** Renyi Yang, Puhua Zeng.

**Methodology:** Renyi Yang, Xiaopeng Yu.

**Resources:** Wei Peng.

**Supervision:** Jincheng Tang, Wei Peng.

**Software:** Xiaopeng Yu.

**Validation:** Hongyao Chen, Wei Peng.

**Visualization:** Hongyao Chen.

**Writing – original draft:** Jingting Zhang, Jincheng Tang.

**Writing – review & editing:** Puhua Zeng.
